# Shift in the submucosal microbiome of diseased peri-implant sites after non-surgical mechanical debridement treatment

**DOI:** 10.3389/fcimb.2022.1091938

**Published:** 2023-01-16

**Authors:** Fei Sun, Yiping Wei, Siqi Li, Yong Nie, Cui Wang, Wenjie Hu

**Affiliations:** ^1^ Department of Periodontology, National Engineering Laboratory for Digital and Material Technology of Stomatology, Beijing Key Laboratory of Digital Stomatology, Peking University School and Hospital of Stomatology, Beijing, China; ^2^ Laboratory of Environmental Microbiology, Department of Energy and Resources Engineering, College of Engineering, Peking University, Beijing, China

**Keywords:** dental implants, peri-implant microbiome, peri-implant diseases, submucosal biofilm, non-surgical mechanical debridement therapy

## Abstract

**Objectives:**

The object of this prospective study was to assess the submucosal microbiome shifts in diseased peri-implant sites after non-surgical mechanical debridement therapy.

**Materials and methods:**

Submucosal plaques were collected from 14 healthy implants and 42 diseased implants before and eight weeks after treatment in this prospective study. Mechanical debridement was performed using titanium curettes, followed by irrigation with 0.2% (w/v) chlorhexidine. Subsequently, 16S rRNA gene sequencing was used to analyze the changes in the submucosal microbiome before and after the non-surgical treatment.

**Results:**

Clinical parameters and the submucosal microbiome were statistically comparable before and after mechanical debridement. The Alpha diversity decreased significantly after mechanical debridement. However, the microbial richness varied between the post-treatment and healthy groups. In network analysis, the post-treatment increased the complexity of the network compared to pre-treatment. The relative abundances of some pathogenic species, such as *Porphyromonas gingivali*s, *Tannerella forsythia*, *Peptostreptococcaceae XIG-6 nodatum*, *Filifactor alocis*, *Porphyromonas endodontali*s, TM7 sp., and *Desulfobulbus* sp. *HMT 041*, decreased significantly following the non-surgical treatment.

**Conclusions:**

Non-surgical treatment for peri-implant diseases using mechanical debridement could provide clinical and microbiological benefits. The microbial community profile tended to shift towards a healthy profile, and submucosal dysbiosis was relieved following mechanical debridement.

## 1 Introduction

Dental implants are a widely accepted treatment option as a replacement for missing teeth. The clinical success of osseointegrated implants is well documented. A prospective study reported satisfactory 10-year survival rates of over 99% at both patients and implants (van Velzen et al., 2015). However, the increased use of dental implants has resulted in an increased incidence of biological complications, also called peri-implant inflamed diseases (Derks and Tomasi, 2015). Peri-implant diseases are further classified as peri-implant mucositis and peri-implantitis. Peri-implant mucositis is characterized by inflammatory lesions of the soft tissues, and peri-implantitis can result in the loss of implant-supported bone beyond the initial bone remodeling (Berglundh et al., 2018; Renvert et al., 2018).

A consensus states that plaque accumulation on implant surfaces is the primary etiological factor associated with the development of peri-implant diseases (Berglundh et al., 2018). Treatment of peri-implant inflammation, either peri-implantitis or peri-implant mucositis, is primarily focused on decontamination of the implant surface to control the infection. The first step is non-surgical therapy, such as mechanical debridement (Renvert et al., 2019). Various peri-implant non-surgical treatment modalities have been described, such as various mechanical debridement methods (curettes, ultrasonic, and air polishing), Er: YAG lasers, photodynamic treatments, and pharmaceutical therapies (chlorhexidine, probiotics, and local or systemic antibiotics) (Galofre et al., 2018; Kormas et al., 2020).

According to previous studies, non-surgical therapy (i.e., air polishing, ultrasonic scaling, or lasers) in the management of peri-implant diseases could result in clinical improvements, such as a decreased propensity for bleeding and a reduction in pocket depth (Sahm et al., 2011; Riben-Grundstrom et al., 2015; Schwarz et al, 2015; Galofre et al., 2018; Hentenaar et al., 2021). However, conflicting results have been reported by studies that have investigated the microbiological changes following mechanical debridement. Persson et al. (2010) demonstrated reduced counts of *Aggregatibacter actinomycetemcomitans*, *Lactobacillus acidophilus*, *Streptococcus anginosus*, and *Veillonella parvula* following mechanical debridement using the checkerboard DNA–DNA hybridization method (Persson et al., 2010). However, most studies have shown that non-surgical treatment approaches fail to reduce bacterial counts or significantly affect the peri-implant microbiota (Persson et al., 2011; Hentenaar et al., 2020; Nie et al., 2020). The difference between clinical improvement and microbiological changes requires a focus on the changes in the peri-implant microbiome profile. Next-generation sequencing of the bacterial 16S rRNA gene has recently provided new insights into the diversity of the oral microbiome associated with peri-implant diseases. Its advantages include the detection of the microbial profile at unprecedented depths. However, there is limited information regarding the composition and shift in the peri-implant microbiome following non-surgical treatment.

The present study aimed to assess the shift in the submucosal microbiome profile using 16S rRNA gene sequencing after non-surgical mechanical debridement to highlight the effects of the treatment on the microbial profile.

## 2 Materials and methods

### 2.1 Study subjects

This study was approved by the Ethics Committee of Peking University School and Hospital of Stomatology (PKUSSIRB-201946080) and registered in the Chinese Clinical Trial Registry (ChiCTR2000031392). Patients who visited the Department of Periodontology at the Peking University School and Hospital of Stomatology between September 2019 and September 2021 were screened for eligibility. Patients older than 18 years of age who were healthy and had at least one functional dental implant were included in the study. The implant was diagnosed with peri-implant disease (peri-implant mucositis or peri-implantitis), or as healthy, according to the new implant condition definitions presented at the 2017 World Workshop (Berglundh et al., 2018). The exclusion criteria were the following: patients who have received any kind of periodontal or peri-implant treatment in the past 6 months; use of systemic antibiotics within the past 6 months; pregnancy or lactation; presence of implant mobility; systemic diseases that affect the treatment effect (uncontrolled diabetes, osteoporosis, and a history of bisphosphonates); and smoking more than10 cigarettes per day. Written informed consent was obtained from all the participants.

### 2.2 Clinical examination and non-surgical treatment

Peri-apical radiographs were obtained using the parallel technique, and the peri-implant bone levels were evaluated. Patients received a full mouth examination one week after supragingival scaling. Periodontal parameters, including pocket probing depth (PPD), bleeding index (BI) (Mazza et al., 1981), and plaque index (PLI) (Silness and Loe, 1964), were recorded at all six sites (mesio-buccal, mid-buccal, disto-buccal, mesio-lingual, mid-lingual, and disto-lingual) for each tooth. All clinical examinations were performed by the same clinician, and the treatment procedure was performed by another clinician.

Before treatment, oral hygiene instructions were provided to improve the plaque control. Under appropriate anesthesia, debridement with titanium curettes (Hu-Friedy, Chicago, IL, USA) and irrigation with 0.12% chlorhexidine were performed for the diseased peri-implant sites. For periodontitis sites, scaling and root planing using an ultrasonic device and a metal curette (Gracey curette, Hu-Friedy, Chicago, IL, USA) were performed before peri-implant mechanical debridement. A re-examination was performed eight weeks after the treatment.

### 2.3 Sample collection

Submucosal microbial samples were obtained from diseased (peri-implant mucositis and peri-implantitis) peri-implant sites and re-sampled eight weeks after the treatment. Submucosal samples from healthy implants were collected as healthy controls. Sample implants were isolated using cotton rolls and air-dried, while supra-mucosal plaques were removed using curettes. Six paper points (ISO #35) were inserted into the bottom of the peri-implant pocket at six different sites and held in place for 30 s. The paper points were placed in a sterile 1.5-mL Eppendorf tube. The samples were transferred as soon as possible to the laboratory. After adding 350 µL of TE buffer (50 mM Tris-HCl, 1 mM EDTA; pH 8.0) to each tube and shaking for 1 h, the samples were centrifuged, and the pellets were separated and stored at -80°C for DNA extraction.

### 2.4 DNA extraction

Microbial DNA was extracted using the QIAamp DNA Mini Kit (QIAGEN, Hilden, Germany) according to the manufacturer’s protocol. A NanoDrop 2,000 UV-VIS spectrophotometer (Thermo Scientific, Wilmington, USA) was used to test the concentration and purification of the final DNA, and 1% agarose gel electrophoresis was used to determine the DNA quality. Primers 338F (5’-ACTCCTACGGGAGGCAGCAG-3’) and 806R (5’-GGACTACHVGGGTWTCTAAT-3’) were used to amplify the V3-V4 hypervariable regions of the bacterial 16S rRNA gene using a thermocycler (GeneAmp 9700, ABI, USA). Based on the quantity and quality of the extracted DNA, the samples were diluted to 1 ng/μL using sterile water and stored at -80°C until use.

### 2.5 Illumina MiSeq sequencing

Purified amplicons were merged into equimolar concentrations and paired-end sequenced (2 × 250) on an Illumina MiSeq platform (Majorbio Bio-Pharm Technology, Shanghai, China). Raw sequencing data were filtered and trimmed using QIIME (version 1.17), and then classified into operational taxonomic units (OTUs) with a 97% similarity cutoff using UPARSE (version 7.1, http://drive5.com/uparse/ ). The taxonomy of each 16S rRNA gene sequence was analyzed using the Ribosomal Database Project Classifier tool (http://rdp.cme.msu.edu /) against the Human Oral Microbiome Database (HOMD V15.2) using a default confidence threshold of 0.7 (Dewhirst et al., 2010). The raw reads were deposited in the NCBI Sequence Read Archive (SRA) database (Accession Number: PRJNA861252).

### 2.6 Bioinformatics and statistical analysis

The mean clinical parameters were tested for normality using the Kolmogorov–Smirnov method, and the pre-treatment and post-treatment data were compared using a paired Student’s *t*-test (peri-implant probing depth, PPD) or Wilcoxon rank-sum test (bleeding index, BI; plaque index, PLI). SPSS version 24 (IBM Corporation, Armonk, NY, USA) was used to perform statistical analyses.

Alpha diversity was assessed using the Chao1 and Shannon indices to estimate the microbial richness and diversity, respectively. All alpha diversity results were compared using the Wilcoxon rank-sum test. Principal component analysis (PCoA) based on weighted UniFrac distance was conducted at the OTU level to examine the similarity in microbial composition between samples (Lozupone et al., 2006; Lozupone et al., 2007). An analysis of similarities (ANOSIM) was conducted to compare the similarities between groups. The correlation of microbes was analyzed using Spearman’s correlation coefficients, and the co-occurrence networks were visualized using Cytoscape 3.5.1. The significance of the differences in relative abundance at the species level between the pre-treatment and post-treatment groups was analyzed using the Wilcoxon rank-sum test. BugBase (https://bugbase.cs.umn.edu/index.html) was used to compare changes in the microbial phenotype.

## 3 Results

### 3.1 Clinical outcomes and microbial profiles of submucosal plaque samples

Twenty-five patients with 42 diseased implants (13 patients with peri-implant mucositis, n = 18; 12 patients with peri-implantitis, n = 24) underwent mechanical debridement. Nine healthy patients with 14 healthy implants were sampled as healthy controls and underwent a clinical examination at baseline. Eight weeks after non-surgical treatment, significant reductions were observed in all clinical parameters (*p* < 0.001). Before the treatment, the mean PPD of the diseased implants was 5.9 mm, which was reduced to 4.7 mm 8 weeks later. Moreover, the mean BI changed from 3.4 to 2.7, and the mean PLI decreased from 1.6 to 0.8. The demographic and clinical parameters of the patients are listed in **Table 1** and **Supplementary Table 1**.

**Table 1:** Demographic and clinical characteristics of study participants.

	Healthy	Diseased	
		Baseline	Week 8
Patient characters			
N (patients)	9	25	
Age (years)	40.2 ± 12.6	52.2 ± 11.0	
Gender (male/female)	2/7	14/11	
Smokers	None	2	
Sampled implants characters			
n (implants)	14	42	
Region (anterior/posterior)	2/12	6/36	
Jaw (maxilla/mandible)	7/7	24/18	
PPD	2.3 ± 0.3	5.9 ± 1.8	4.7 ± 1.5*
BI	0	3.4 ± 0.7	2.7 ± 0.8*
PLI	0.4 ± 0.7	1.6 ± 0.9	0.8 ± 0.6*

PPD, peri-implant probing depth; BI, bleeding index; PLI, plaque index.

*p < 0.001 by Mann–Whitney U test between baseline and week 8.

Sequencing of submucosal biofilm samples (n = 98) produced 49,47,203 sequences, which corresponded to an average length of 420 bp. Overall, 1673 OTUs were detected and classified into 16 phyla, 38 classes, 65 orders, 118 families, 238 genera, and 477 species. The rarefaction curve assessment indicated that the number of reads obtained was sufficient for microbiological analysis (**Supplementary Figure 1**).

### 3.2 Compositions in submucosal bacterial communities

**Figure 1:**
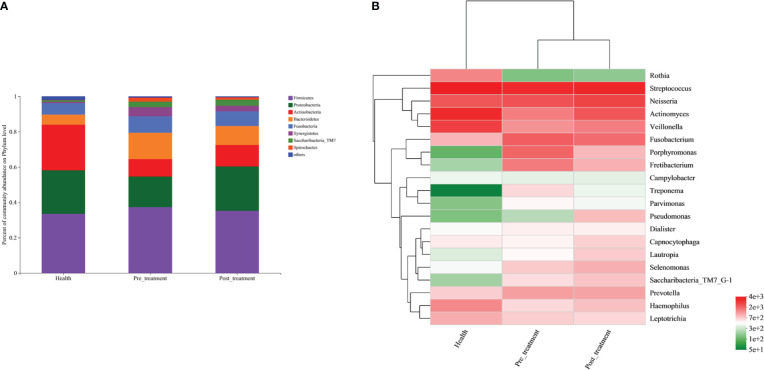
Compositions of the submucosal bacterial communities. **(A)** Relative prevalence of bacteria at the phylum level in healthy, pre-treatment, and post-treatment groups. **(B)** A heatmap showing the distribution of the top 20 genera across the three groups.

The respective distributions of the top eight phyla are presented in a bar plot (**Figure 1A**). The prevalence of *Firmicutes, Bacteroidetes, Fusobacteria, Synergistetes* and *Spirochaetes* was highest in the pre-treatment group and decreased in the post-treatment group, while the least prevalence was observed in the healthy group. *Actinobacteria* were enriched in the healthy group and were impoverished in the pre-treatment group. After non-surgical mechanical treatment, the proportion of *Actinobacteria* increased. The distribution of the top 20 genera is presented in a heat map (**Figure 1B**). We found that some well-recognized pathogenic genera, such as *Fusobacterium, Porphyromonas, Fretibacterium, Prevotella, Treponema*, and *Parvimonas*, were predominant in the pre-treatment group. Meanwhile, these genera showed lower relative prevalences in the post-treatment and healthy groups. Some genera, such as *Rothia, Streptococcus, Actinomyces, Veillonella, Haemophilus*, and *Leptotrichia*, had the highest relative prevalence in the healthy group, followed by the post-treatment group, and the pre-treatment group.

### 3.3 A shift in submucosal microbiome after mechanical debridement

**Figure 2:**
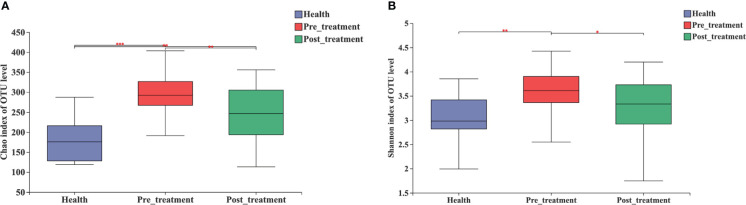
Alpha diversity estimates for the microbial profiles in the healthy, pre-treatment, and post-treatment groups. **(A)** Microbial richness presented by Chao1. **(B)** Microbial diversity presented by Shannon. *** *p* < 0.0001, ** *p* < 0.01, * *p* < 0.05 by Wilcoxon rank-sum test.

The Chao1 and Shannon indices of the microbial community were the highest in the pre-treatment group and significantly decreased after non-surgical mechanical debridement (**Figure 2A**). A slightly higher alpha diversity was observed in the post-treatment group than that in the healthy group. However, a significant difference was detected only in the Chao1 index (**Figures 2A, B**).

**Figure 3:**
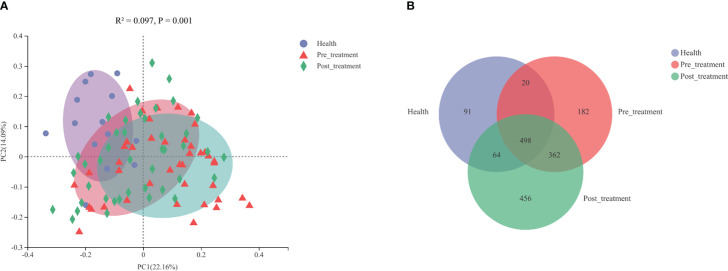
**(A)** Principal coordinated analysis (PCoA) plots constructed based on weighted Unifrac distances at the OTU level. **(B)** A venn diagram representing the shared and unique OTUs in the submucosal microbiomes of the healthy, pre-treatment, and post-treatment groups.

The PCoA results revealed that the bacterial profiles of the three groups were dissimilar (R^2^ = 0.0966, *p* = 0.001, ANOSIM, **Figure 3A**). A Venn diagram was constructed to show the number of different OTUs that were common and unique to the submucosal samples in the three groups. The pre-treatment/healthy and post-treatment/healthy groups shared common OTUs, which increased from 518 to 562 (**Figure 3B**).

### 3.4 The inter-microbial interactions

**Figure 4:**
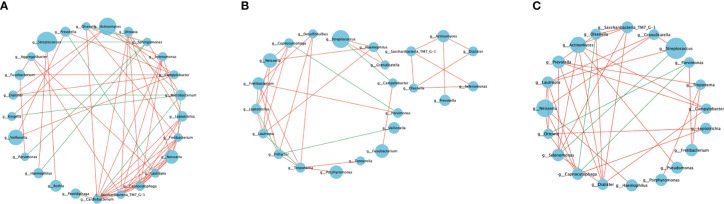
Microbial co-occurrence networks. The co-occurrence network of the genera with relative abundance >1% (|SpearmanCoef. >0.5 and *p* < 0.05). **(A)** Bacterial interactions in the healthy group. **(B)** Bacterial interactions in the pre-treatment group. **(C)** Bacterial interactions in the post-treatment group. The node size represents the abundance of each taxon. The edge color represents positive (red) and negative (green) correlations between the two genera.

Network analysis was performed at the genus level to evaluate the polymicrobial interactions between the taxa. Only the major genera (relative prevalence >1%) exhibited a significant correlation (*p* < 0.05) with absolute values of the correlation coefficient >0.5 (**Figure 4**).

Each network was dominated by several positive and several negative correlations. The highest number of shared nodes and links was found in the co-occurrence network of the healthy group, which contained 25 nodes and 57 links. The networks of the pre-treatment and post-treatment groups consisted of 23 nodes, 34 links, 20 nodes, and 36 links, respectively. The average degree (average links per node) represents the microbial interaction complexity (Ya et al., 2021). The order of decreasing degree according to network complexity was healthy controls (2.28) > post-treatment group (1.8) > pre-treatment group (1.48) (**Table 2**). The network in the healthy group had more interacting taxa, various co-occurrence and mutual exclusion interactions, and a more stable bacterial community. The network of the pre-treatment group comprised the sparsest bacterial links, which indicated an unstable bacterial community. The network of the post-treatment group showed increased polymicrobial interactions and complexity after mechanically non-surgical treatment.

**Table 2:** Topological properties of interaction networks.

Network property	Healthy controls	Pre-treatment	Post-treatment
Nodes	25	23	20
Links	57	34	36
Average degree	2.28	1.48	1.8
Transitivity	0.594	0.387	0.443

Average degree: average links per node.

The genera *Tanneralla*, *Fillifactor*, and *Desulfobulbus* were only found in the network of the pre-treatment group (**Figure 4B**). While the genera *Porphyromonas*, *Treponema*, and *Granulicatella* were only found in the network of the diseased peri-implant sites (**Figures 4B, C**). The genera *Rothia, Microbacterium, Peptidiphaga, Sphingomonas, Kingella, Cardiobacteium*, and *Agrregatibacter* were only found in the network of the healthy group (**Figure 4A**).

### 3.5 Comparison of submucosal bacterial types and microbiome phenotypes before and after mechanical debridement

**Figure 5:**
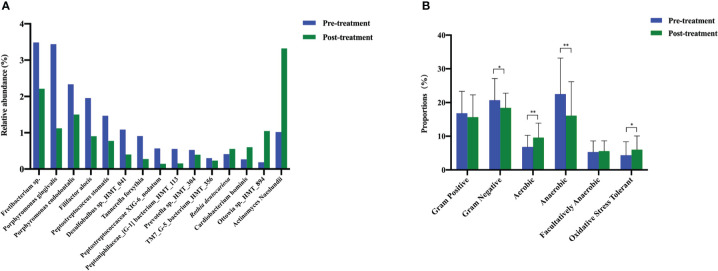
The changes in the bacterial types and microbiome phenotypes after mechanical non-surgical treatment. **(A)** Microbes with significant shifts after mechanical non-surgical treatment. **(B)** Microbiome phenotype prediction by Bugbase. ** *p* < 0.01, * *p* < 0.05 by Wilcoxon rank-sum test.

To evaluate the microbiological outcomes after peri-implant nonsurgical treatment, we compared the relative prevalence of various taxa before and after treatment. The top 15 significantly different species are listed in **Figure 5A**. We found 11 species that decreased significantly, and 4 species that increased significantly after mechanical debridement.

According to BugBase, the prevalence of aerobic bacteria increased significantly and that of gram-negative and anaerobic bacteria decreased significantly after non-surgical treatment. The proportion of gram-positive bacteria decreased slightly, and that of facultatively anaerobic bacteria increased slightly; however, no significant differences were detected. Mechanical debridement significantly improved the oxidative stress tolerance of the submucosal microbiome (**Figure 5B**).

## 4 Discussion

This study involved a comparative analysis of the changes in peri-implant submucosal microbiota following non-surgical mechanical debridement therapy. Our results revealed that mechanical debridement tended to shift the microbial profile toward healthy profiles. This emphasizes the importance of peri-implant non-surgical mechanical debridement from both clinical and microbiological perspectives.

With respect to the clinical parameters, PPD, BI, and PLI decreased significantly after mechanical debridement, while the aforementioned parameters did not reach the healthy levels. These findings coincide with those of previous studies, which showed limited improvements in clinical parameters following mechanical nonsurgical treatment. (Renvert et al., 2009; Schwarz et al., 2015; John et al., 2017). Regarding microbial diversity, we used Chao 1 to estimate the microbial richness (number of species) and the Shannon index to measure the evenness (referring to the similarity in the prevalences of different species in the microbiome) of species. Both parameters decreased significantly after the non-surgical treatment. However, a statistically significant difference persisted in Chao 1, but not in the Shannon index between the post-treatment and healthy groups. The unchanged level of microbial richness may provide a microbiological reason for the limited clinical effects. To achieve better peri-implant inflammation resolution, adjunctive therapy during mechanical debridement might provide benefits to further reduce the total number of species.

Peri-implant diseases are associated with dysbiosis of the submucosal microbiome. Previous studies have found that disease severity (deeper pocket depth and increased marginal bone loss) is correlated with peri-implant submucosal microbiome dysbiosis (Kröger et al., 2018; Lu et al., 2021; Shi et al., 2022). Some studies have demonstrated that inflamed peri-implants are associated with increased microbial diversity compared with those in healthy implants (Zheng et al., 2015; Sanz-Martin et al., 2017). According to Lu et al. (2021), the increase in microbial diversity partly revealed the dysbiosis of the microbiome along with the onset of peri-implant inflammation (Lu et al., 2021). Furthermore, our results revealed that inflamed peri-implants had higher microbial diversity and microbiome dysbiosis levels than those of healthy implants.

In the present study, we found that mechanical debridement could alter the submucosal peri-implant microbiome. PCoA results revealed that the health structures in the pre-treatment and post-treatment groups were dissimilar. More common OTUs were shared by the healthy and post-treatment groups than those shared by the healthy and pre-treatment groups, and the distribution of the phyla and some genera in the post-treatment group was similar to that in the healthy group. The aforementioned microbiological results indicated that the microbial profile tended to shift towards a healthy profile and that submucosal dysbiosis was relieved as a result of mechanical debridement.

However, our results are in contrast to those of Nie et al. (2020). Using pyrosequencing, they demonstrated that mechanical debridement could not efficiently alter the submucosal microbiomes in diseased implants. The discrepancies may be due to differences in the methods used (i.e., diagnosis of peri-implant diseases and health conditions, clinical examination, sample collection, DNA extraction, sequencing, and treatment protocols).

Traditionally, the microbiomes in peri-implant diseases are considered similar to those in periodontal diseases, and the translocation of periodontal pathogens around an implant is considered a critical etiology (Lee et al., 1999; Botero et al., 2005; Zhang et al., 2015). According to previous studies, the periodontal microbiome showed significant changes before and after non-surgical periodontal treatment (Chen et al., 2018; Liu et al., 2018). The aforementioned studies revealed an overall trend of decreasing disease-associated taxa and increasing health-associated taxa after the treatment. Our findings add to the knowledge that the peri-implant microbiomes share common features with the periodontal microbiome. In the current study, we also found a similar paradigm of microbial shift after peri-implant non-surgical treatment. Some well-recognized periodontal pathogens, such as *Porphyromonas gingivalis* and *Tannerella forsythia*, decreased significantly following mechanical debridement. Some studies found that *Filifactor alocis*, *Porphyromonas endodontalis*, *TM7* sp., and *Desulfobulbus* sp. *HMT 041*, which have significant virulence properties, may result in inflammatory processes (Aruni et al., 2015; Marchesan et al., 2016; Ma et al., 2017; Sanz-Martin et al., 2017; Bor et al., 2019). As newly proposed periodontal or peri-implant disease-related pathogens, we also found that the prevalence of these lesser-known pathogens decreased significantly after non-surgical treatment. However, the relative prevalence of another red complex member (*Treponema denticola*) and most species in the orange complex (*Fusobacterium nucleatum*, *Parvimonas micra*, and *Campylobacter rectus*) did not significantly vary after the treatment. These species may have the ability to endure mechanical treatment; further studies are warranted to further analyze the characteristics of these bacteria.

Previous studies have compared the microbiomes of healthy and diseased implants and identified genera *Rothia*, *Streptococcus*, *Neisseria*, *Haemophilus*, *Actinomyces*, *Atopobium*, *Gemella*, *Kingella*, *Leptotrichia*, *Propionibacterium*, and *Capnocytophaga* to be mostly associated with healthy implants (Koyanagi et al., 2010; Kumar et al., 2012; da Silva et al., 2014; Zhang et al., 2015; Sanz-Martin et al., 2017; Al-Ahmad et al., 2018; Daubert et al., 2018). In contrast, genera *Fusobacterium, Treponema, Porphyromonas*, *Filifactor*, *Fretibacterium*, *Tannerella*, *Campylobacter, Eubacterium*, *Chloroflexi, Veillonella*, *Tenericutes*, *Synergistetes*, *Desulfobulbus*, *Dialister*, and *Mitsukella* were predominantly found in diseased dental implants (Mombelli and Décaillet, 2011; Kumar et al., 2012). These findings are consistent with the present findings. Moreover, in our study, we found that *Rothia dentocariosa*, *Cardiobacterium hominis*, *Ottowia* sp. *HMT 894*, and *Actinomyces naeslundii* significantly increased post-treatment. These species may play active roles in maintaining peri-implant health and homeostasis.

The present longitudinal study provides a more comprehensive picture of the peri-implant microbiome shift following mechanical debridement rather than focusing on the changes in a few bacteria. However, this study had a few limitations. First, the inclusion of multiple implants recruited from the same patient might have led to a bias. Second, we performed bioinformatics analysis on diseased implants and did not differentiate peri-implant mucositis from peri-implantitis, which might have missed the effects of bone resorption on the submucosal microbiome. Shi et al. reported that bone resorption is correlated with submucosal dysbiosis (Shi et al., 2022). However, it should be pointed out that the classification of peri-implant conditions was based on clinical examinations. Furthermore, though we compared the similarities (ANOSIM) of the microbiomes in peri-mucositis and peri-implantitis at baseline, our results showed that there were no significant differences (R^2^ = 0.0715, *p* = 0.055) in the microbial composition between peri-implant mucositis and peri-implantitis (**Supplementary Figure 2**). The fact that no microbiological differences were statistically significant between peri-implant mucositis and peri-implantitis, might have to do with the mild nature of peri-implantitis in the present patient sample. Third, the participants included smokers. Cigarette smoking is associated with peri-implant diseases (Heitz-Mayfield and Salvi, 2018; Schwarz et al., 2018) and may negatively influence the peri-implant microbiome (Tsigarida et al., 2015; Pimentel et al., 2018).

In conclusion, within the limitations of the present study, we provide new insights into the microbial shifts occurring in diseased peri-implant sites following non-surgical treatment. Mechanical debridement can provide short-term clinical and microbiological benefits in the treatment of peri-implant inflammation.

## Data availability statement

The datasets presented in this study can be found in online repositories. The names of the repository/repositories and accession number(s) can be found below: https://www.ncbi.nlm.nih.gov/ , PRJNA861252.

## Ethics statement

The studies involving human participants were reviewed and approved by Ethics Committee of Peking University School and Hospital of Stomatology. The patients/participants provided their written informed consent to participate in this study.

## Author contributions

WH and CW: conception and design. FS, YW, SL, CW, and WH: recruitment of patients and collection of oral samples. FS and SL: analysis and interpretation of data. FS and YW: manuscript preparation. FS, CW, WH, and YN: manuscript revisions. All authors contributed to the article and approved the submitted version.
