# Supplementary figures and images for: Shift in the submucosal microbiome of diseased peri-implant sites after non-surgical mechanical debridement treatment
Source: Front Cell Infect Microbiol. 2023 Jan 16;12:1091938. doi: 10.3389/fcimb.2022.1091938 (PMC9884694; doi:10.3389/fcimb.2022.1091938)

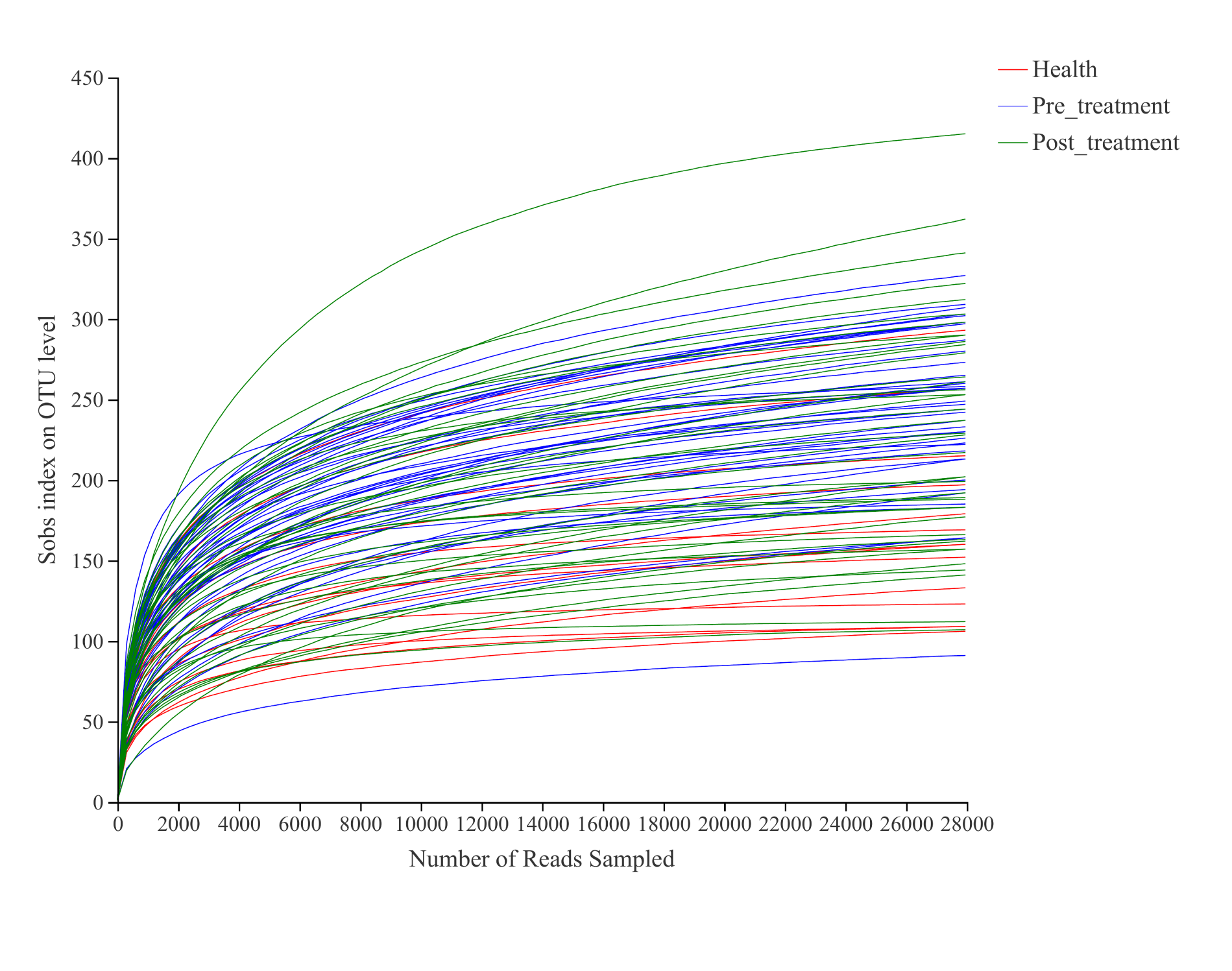

Supplement: Supplementary file 1 [file Image_1.tif]

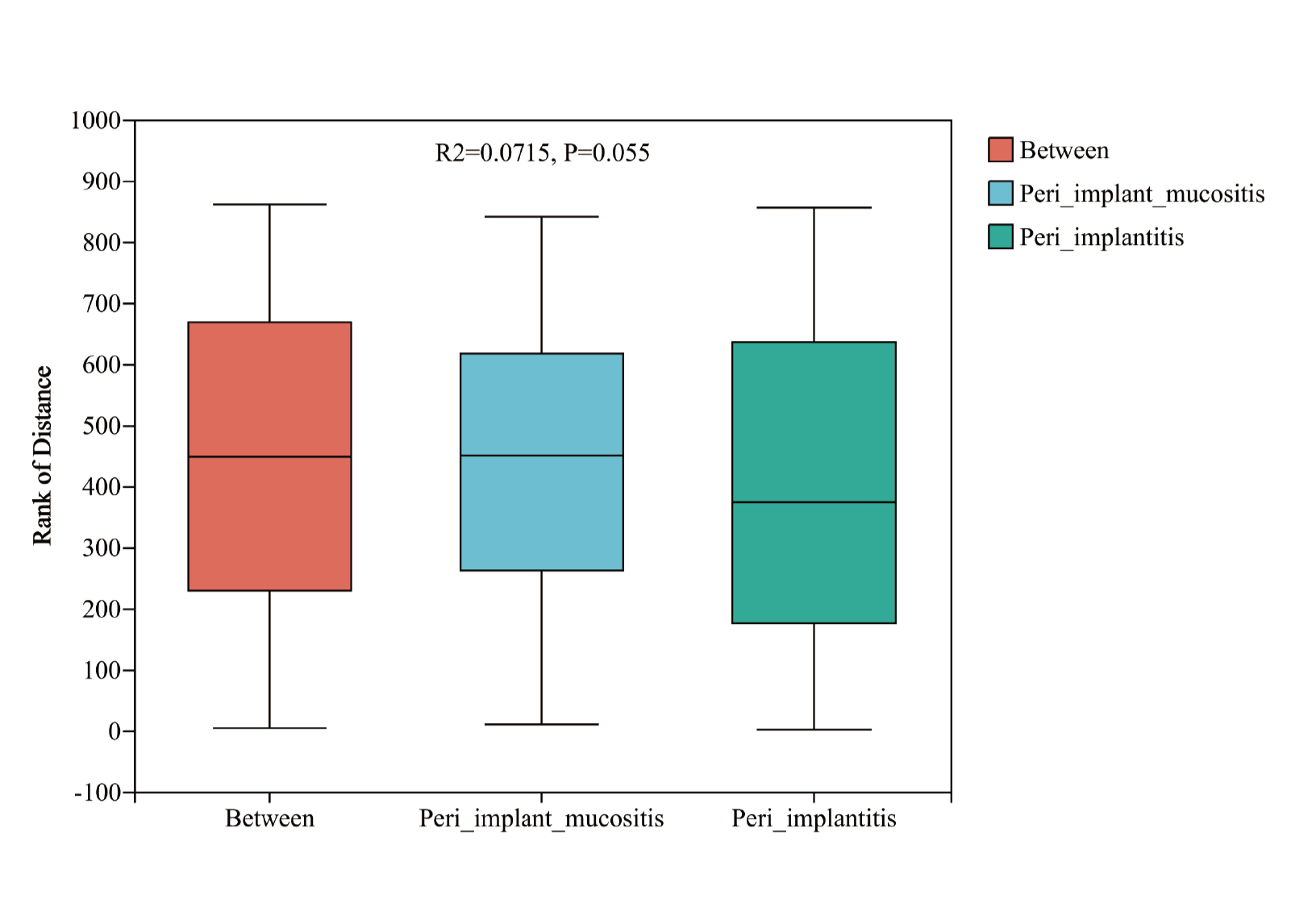

Supplement: Supplementary file 2 [file Image_2.tif]
