# Supplementary material for: Shift in the submucosal microbiome of diseased peri-implant sites after non-surgical mechanical debridement treatment
Source: Front Cell Infect Microbiol. 2023 Jan 16;12:1091938. doi: 10.3389/fcimb.2022.1091938 (PMC9884694; doi:10.3389/fcimb.2022.1091938)
Supplement: Supplementary file 3 [file Table_1.docx]

Supplementary Material

**Supplementary Figure 1** Rarefaction curves calculated by Sobs index.

**Supplementary Table 1** Characteristics of diseased implants

|  | Peri-implant mucositis | | Peri-implantitis | | |
| --- | --- | --- | --- | --- | --- |
|  | Baseline | Week 8 | Baseline | | Week 8 |
| Patient characteristics |  | |  | | |
| N (patients) | 13 | | 12 | | |
| Age (years) | 53.2 ± 11.4 | | 51.2 ± 10.54 | | |
| Gender (male/female) | 7/6 | | 8/4 | | |
| Smokers | 1 | | 1 | | |
| Sampled implant characteristics |  |  |  | | |
| n (implants) | 18 | | 24 | | |
| Region (anterior/posterior) | 4/14 | | 2/22 | | |
| Jaw (maxilla/mandible) | 11/7 | | 13/11 | | |
| PPD | 4.6 ± 0.8 | 3.9 ± 0.8 | 6.9 ±1.7 | 5.4 ± 1.5 | |
| BI | 3.3 ± 0.7 | 2.3 ± 1.0 | 3.5 ± 0.6 | 2.9 ± 0.5 | |
| PLI | 1.5 ± 0.7 | 0.7 ± 0.7 | 1.6 ± 1.1 | | 0.9 ± 0.6 |
| Bone loss | 0 | - | 3.8 ± 1.5 | | - |

PPD, peri-implant probing depth; BI, bleeding index; PLI, plaque index.

**Supplementary Figure 2** ANOSIM between peri-implant mucositis and peri-implantitis at baseline.
